# Fuzzy method for pre-diagnosis of breast cancer from the Fine Needle Aspirate analysis

**DOI:** 10.1186/1475-925X-11-83

**Published:** 2012-11-02

**Authors:** Gláucia RMA Sizilio, Cicília RM Leite, Ana MG Guerreiro, Adrião D Dória Neto

**Affiliations:** 1 Department of Computer Engineering and Automation - DCA and Department of Biomedical Engineering, Federal University of Rio Grande do Norte (UFRN), Caixa Postal 1524 – Campus Universitário - UFRN/CT/DCA, Rio Grande do Norte, CEP 59072-970, Brazil; 2 Department of Informatic - State University of Rio Grande do Norte (UERN), Av. Campus Universitário, Rio Grande do Norte, BR 110, Km 46, CEP 59625-620, Brazil

**Keywords:** Computational intelligence, Fuzzy logic, Fine needle aspirate, Decision support system, Breast cancer diagnosis, Telediagnosis

## Abstract

**Background:**

Across the globe, breast cancer is one of the leading causes of death among women and, currently, Fine Needle Aspirate (FNA) with visual interpretation is the easiest and fastest biopsy technique for the diagnosis of this deadly disease. Unfortunately, the ability of this method to diagnose cancer correctly when the disease is present varies greatly, from 65% to 98%. This article introduces a method to assist in the diagnosis and second opinion of breast cancer from the analysis of descriptors extracted from smears of breast mass obtained by FNA, with the use of computational intelligence resources - in this case, fuzzy logic.

**Methods:**

For data acquisition of FNA, the Wisconsin Diagnostic Breast Cancer Data (WDBC), from the University of California at Irvine (UCI) Machine Learning Repository, available on the internet through the UCI domain was used. The knowledge acquisition process was carried out by the extraction and analysis of numerical data of the WDBC and by interviews and discussions with medical experts. The PDM-FNA-Fuzzy was developed in four steps: 1) Fuzzification Stage; 2) Rules Base; 3) Inference Stage; and 4) Defuzzification Stage. Performance cross-validation was used in the tests, with three databases with gold pattern clinical cases randomly extracted from the WDBC. The final validation was held by medical specialists in pathology, mastology and general practice, and with gold pattern clinical cases, i.e. with known and clinically confirmed diagnosis.

**Results:**

The Fuzzy Method developed provides breast cancer pre-diagnosis with 98.59% sensitivity (correct pre-diagnosis of malignancies); and 85.43% specificity (correct pre-diagnosis of benign cases). Due to the high sensitivity presented, these results are considered satisfactory, both by the opinion of medical specialists in the aforementioned areas and by comparison with other studies involving breast cancer diagnosis using FNA.

**Conclusions:**

This paper presents an intelligent method to assist in the diagnosis and second opinion of breast cancer, using a fuzzy method capable of processing and sorting data extracted from smears of breast mass obtained by FNA, with satisfactory levels of sensitivity and specificity. The main contribution of the proposed method is the reduction of the variation hit of malignant cases when compared to visual interpretation currently applied in the diagnosis by FNA. While the MPD-FNA-Fuzzy features stable sensitivity at 98.59%, visual interpretation diagnosis provides a sensitivity variation from 65% to 98% (this track showing sensitivity levels below those considered satisfactory by medical specialists). Note that this method will be used in an Intelligent Virtual Environment to assist the decision-making (IVEMI), which amplifies its contribution.

## Background

Breast cancer is one of the leading causes of death among women worldwide and it is confirmed that early detection and accurate diagnosis of this disease can ensure long-term patient survival [1]. According to the World Health Organisation [2], about one third of the costs of cancer treatment can be reduced if cases are detected and treated early.

On the other hand, aiming to provide greater security, reliability and robustness to services and procedures, mainly when dealing with human lives, healthcare processes are increasingly becoming computerized. A growing area of research relates to the use of techniques from Computational Intelligence (CI) applied to the processing of information necessary for the medical diagnosis. We can cite as examples, [3-15].

This paper presents a method to assist in breast cancer diagnosis from the analysis of descriptors extracted from smears of breast mass obtained by FNA (Fine Needle Aspirate), incorporating features of computational intelligence (in this case, fuzzy logic) and inserted into a collaborative telediagnosis environment (called IVEMI - [16]).

### Diagnosis of breast cancer and FNA

A carcinogen breast tumor is a breast mass that is growing abnormally and uncontrolled. There are three popular methods for breast cancer diagnosis: mammography; FNA with visual interpretation; and surgical biopsy [17]. The ability of these methods to diagnose cancer correctly when the disease is present is: mammogram - from 68% to 79%; FNA with visual-interpretation - from 65% to 98%; and surgical biopsy - 100%. [18]. It is noted that: mammography lacks sensitivity; the sensitivity of FNA with visual interpretation varies greatly (as a result of the visual interpretation); and although surgical biopsy is accurate it is also a very intrusive, time-consuming and expensive method [19].

FNA, which has been widely accepted in the approach to investigating mammary lesions, is the easiest and fastest biopsy technique to be performed, being a percutaneous procedure (through the skin) in which the specialist physician uses a thin needle (which varies from 0.6 to 0.8 mm) and a syringe to take samples of fluid from a breast cyst or remove clusters of cells in a solid mass. The needle is inserted into the skin toward the lesion, with the objective of collecting cells for further evaluation of their morphology, quantity and distribution through cytological examination.

The genetic material extracted from the breast by FNA is usually sent to a Pathology laboratory for examination by pathologists (doctors specialized in disease diagnosis through lab testing), who perform the analysis identifying the cells’ characteristics from observing, under a microscope, smears made with this material on sheets of glass and stained using special techniques.

### Computational intelligence and fuzzy logic

Computational Intelligence (CI) enables, through intelligent techniques some of them inspired by nature, the development of intelligent systems that imitate aspects of human behaviour, such as: learning, perception, reasoning, evolution and adaptation [20]. Some examples of Computational Intelligence techniques are: Artificial Neural Networks, biological neuron-inspired technique [14,15]; Evolutionary Computation, inspired by biological evolution [12]; Expert Systems, inspired by inference process [11]; and Fuzzy Logic, inspired by language processing.

The fuzzy systems theory is a formal approach that aims to address the modelling, representation, reasoning and the inaccurate information procedure as a troubleshooting strategy [21].

Introduced in 1965 [22], the fuzzy set theory is a tool to model the imprecision and ambiguity that arises in complex systems [22,23], and it was created from the combination of the concepts of classical logic and groupings of Łukasiewicz [24] defining degrees of relevance.

A fuzzy set differs from a classic set to assign to each element a value in the unit interval [0, 1]. Specifically, a fuzzy set is defined as a function A of a set x, called universe of discourse, to [0, 1]. The function A is referred to as a membership function, and the value A(x) represents the degree of relevance – or compatibility – of the element x with the concept represented by all the fuzzy set. Thus, the fuzzy logic proposed by Zadeh [22,23] provides a mathematical model for the processing of inaccurate or vague information and concepts, intending to make computers carry out inferences as people.

**Figure 1:**
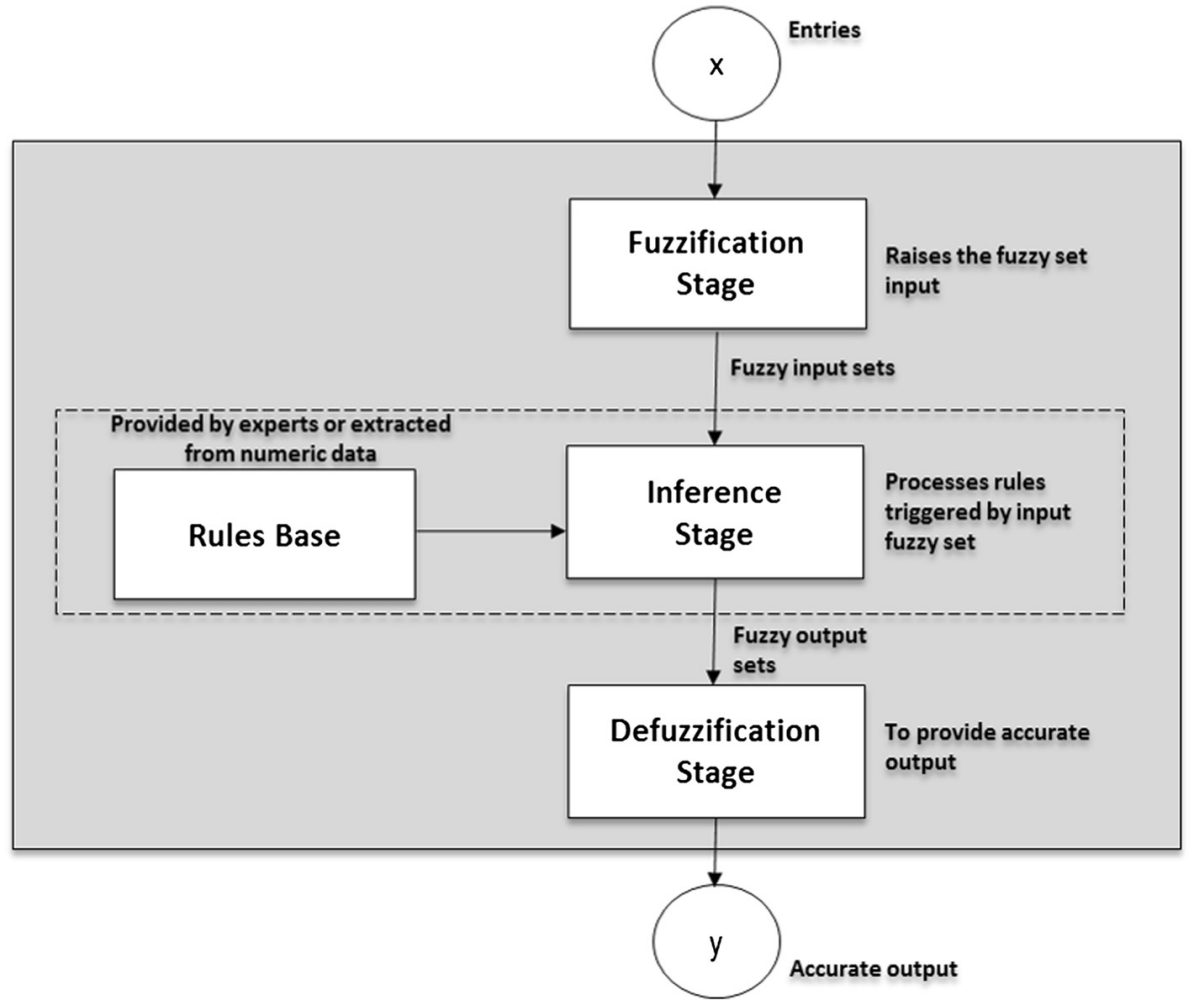
Structure of a Fuzzy System Process.

The fuzzy processing is generally composed of: Rules Base (provided by specialists or extracted from numerical data); Fuzzification Stage (it activates the rules from a set of precise entries); Inference Stage (determines how rules are enabled); Defuzzification Stage (it provides precise output, generating a fuzzy set of output), as illustrated in Figure 1.

## Methods

**Figure 2:**
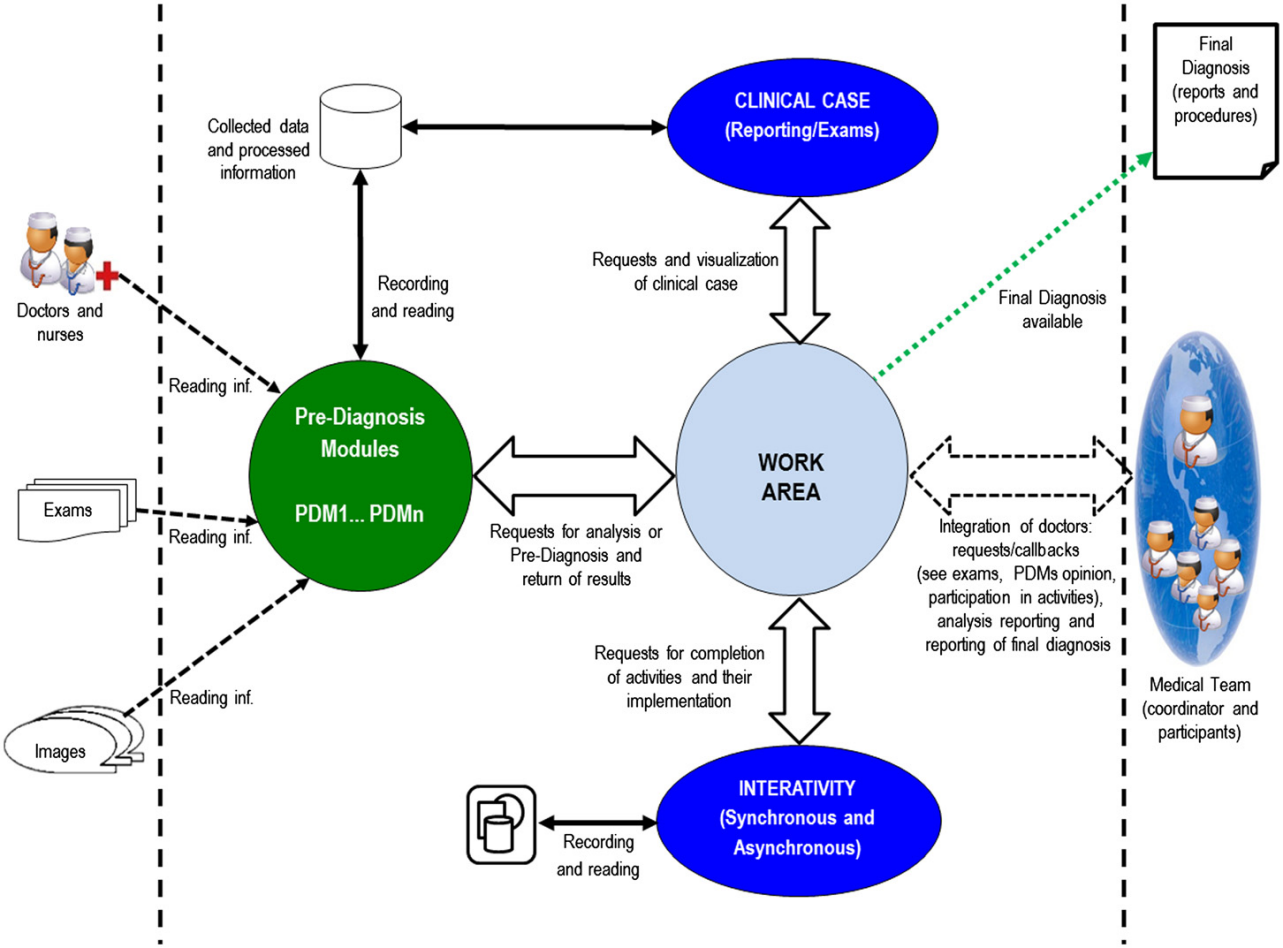
Architecture of IVEMI.

This fuzzy method that assists in the diagnosis and second opinion of breast cancer, called Pre-Diagnosis Module FNA-Fuzzy (PDM-FNA-Fuzzy), was developed through the analysis of extracted smears from breast mass obtained by FNA. The PDM-FNA-Fuzzy is inserted into the Intelligent Virtual Environment of Medical Interaction (IVEMI), which is a virtual environment (architecture shown in Figure 2) from which the doctor responsible can trigger, requesting pre-diagnosis or second opinion on clinical cases with suspected breast cancer and whose patient has undergone the examination of FNA.

### Data acquisition

For FNA data acquisition, the Wisconsin Diagnostic Breast Cancer Data (WDBC), of UCI Machine Learning Repository, available on the internet by the domain of University of California Irvine [25] was used. The WDBC is a public database, consisting of a gold pattern data set^1^, i.e. with confirmation of malignant and benign diagnosis.

The selected database, WDBC, was created in 1993, and presents 569 records of patients with known diagnosis (357 cases being benign and 212 cases malignant) and uses material (smears) collected by FNA, transformed into a digital image from which the main parameters (descriptors) were extracted.

For viewing and manipulating data from WDBC we used MATLAB (MathWorks – student version).

### Pre-processing of data

**Figure 3:**
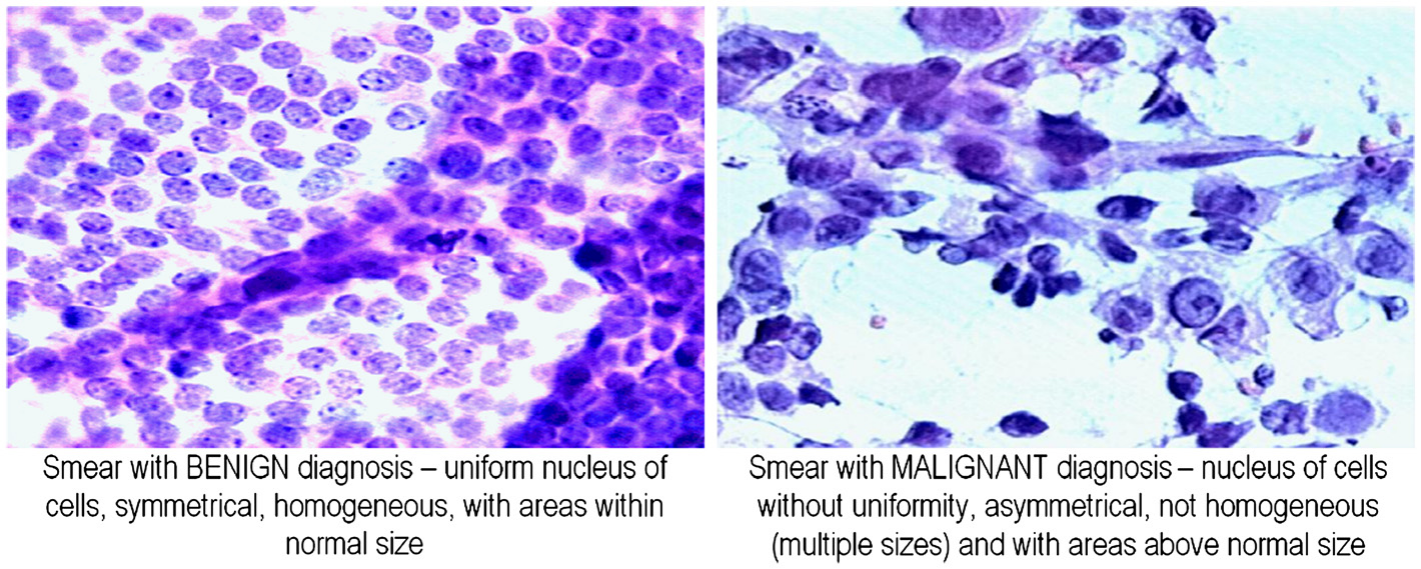
Captured images of layers of glass with smears of breast mass obtained by FNA (the parts stained correspond to cell nuclei).

Samples (small droplets of viscous liquid) were collected from aspiration of breast mass with thin needle, spread on glass blades slides, stained (aiming to highlight the cell nuclei) and digitized [26]. Examples of captured images are presented in Figure 3. Then, in the process of digital evaluation performed by Street et al. [26], the exact location of each cell nucleus was specified, and the morphometric analysis of the cell nuclei, extracting characteristics such as size, shape and texture was complete.

In addition to the code for the identification and diagnosis (gold pattern)^1^, each record of WDBC presents 10 descriptors (related to the cell nucleus and modelled such that the highest values are associated with malignancy): radius; texture; perimeter; area; smoothness; compactness; concavity; concave points; symmetry; and fractal dimension. We must point out that the mean value, the extreme value and the standard error of each descriptor were calculated for each image, resulting in a total of 30 (thirty) resources for each case in the study.

The knowledge acquisition process was accomplished in two ways: (i) extraction and analysis of numerical data of WDBC, considering the same as gold pattern (i.e. with diagnosis confirmed)^1^, and (ii) interviews and discussions with medical experts^2^ (of pathology, general practitioner and mastology areas) who provided technical support and followed the development of this Pre-Diagnosis Intelligent Method.

To reduce the dimensionality of the problem and optimize the processing tests, the PCA^3^ technique in WDBC was applied (average values), once having verified that the descriptors with higher energy rates are, in decreasing order: area, perimeter, texture, and radius. The experiment was repeated for the extreme values, obtaining the same result, and the analyses carried out were confirmed with medical specialists (of pathology, general practitioner and mastology areas).

**Figure 4:**
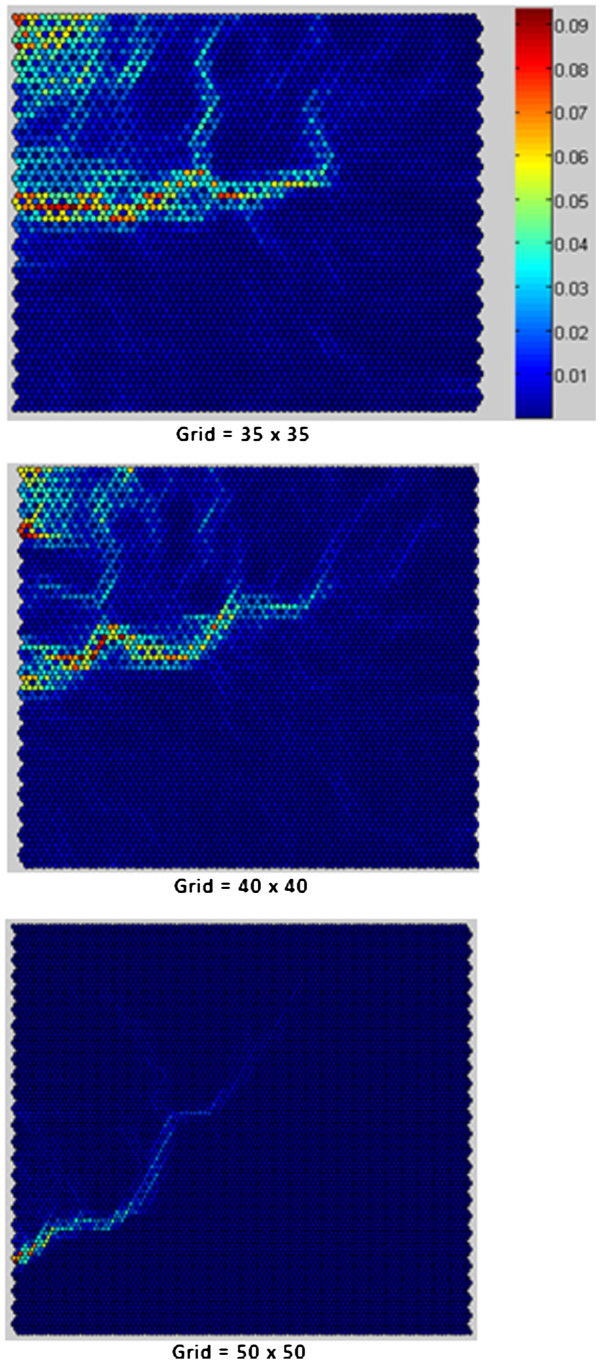
SOM applied to visualization pattern matches to high-dimensional data of WDBC.

Aimed at the collation and visualization of high-dimensional data, after normalization of descriptors, the SOM^4^ algorithm was applied with: linear initialization; hexagonal topology; gaussian neighborhood function; neighborhood radius equal to 1; in a 10-dimensional space of characteristics (descriptors); and with variations in the size of the grid and the amount of iterations of the algorithm batch type. The results of the application of SOM were viewed using the Unified distance Matrix (U-Matrix) [27], a technique that presents weight-distance relationships between neighboring neurons of output space (distance between the units of the grid), showing a separation between the groups (classes of patterns). In the U-Matrix, relations between the neighboring neurons are seen on the surface U (x, y) as "valleys" and "mountains". Valleys - the topographic relief - correspond to the regions of neurons that are similar, while mountains, i.e. relatively high values in the U-matrix, reflect the dissimilarity between neighboring neurons and may be associated with borders of groups of neurons [28]. The topological order property of the SOM, as shown in the right side scale in Figure 4, in the U-Matrix is as folows: a dark blue color represents that the distance between the nodes (units) is small ("valleys") and thus classes of patterns exist; the light blue, green and yellow indicate an average distance between the nodes (beginning of the “mountains”); the orange and red represent that there is a great distance between the nodes ("mountains"), i.e. they are gaps that serve to separate the classes. The application of the SOM for visualization of high-dimensional data (Figure 4) showed that it is not possible to obtain distinct groups (classes of patterns), indicating the existence of nebulous data groupings in the WDBC, which justifies the use of Fuzzy logic in the method developed.

Parallel to the application of PCA and SOM and, mainly, through preliminary analysis of the WDBC descriptors and related images performed along with medical specialists (in pathology, general practice and mastology), were: a) the extracted (selected) descriptors that was more relevant to the diagnosis of breast cancer from the analysis of cell nuclei of smears obtained by FNA, the most relevant being, the "area", the "perimeter" and the "texture"; b) the discarded descriptors, "fractal dimension", "compactness" and "concavity", because they are not actually used in medical practice for pathological analysis; and c) newly generated descriptors, in order to translate the method evaluations normally carried out by pathologists and that were not directly presented in WDBC.

Among the newly generated descriptors, those that presented a significant influence on the results “improvement” were the descriptors: “uniformity”, difference between the radius extreme value and the radius mean value, representing whether the cellular nuclei have similar or highly variable sizes; and “homogeneity”, difference between the extreme value of symmetry and the mean value of symmetry, representing whether the cellular nuclei have similar or highly variable symmetries.

Complementing the analysis of the numerical data, the minimum and maximum parameters for each diagnosis known (benign and malignant) of each descriptor used in the development of PDM-FNA-Fuzzy (as shown in Table 1) were detected, excluding the descriptors discarded by medical specialists. Results showed the existence of fuzzy intervals for all descriptors (benign GPD values are within the range of the malignant GPD and vice versa), not being linearly possible to diagnose breast mass as benign or malignant.

**Table 1:** Minimum and maximum parameters for each diagnosis (benign and malignant) of each descriptor

**DESCRIPTOR**	UNIT**	GPD^*^BENIGN		GPD^*^MALIGNANT	
		**Minimum Value**	**Maximum Value**	**Minimum Value**	**Maximum Value**
Area	μm^2^	143.5	992.1	361.6	2501
Perimeter	μm	43.79	114.6	71.9	188.5
Texture	dimensionless	9.71	33.81	10.38	39.28
Radius	μm	6.981	17.85	10.95	28.11
Smoothness	μm	0.05263	0.1634	0.07371	0.1447
Concave Points	quantity	0	0.08534	0.02031	0.2012
Simetry	μm	0.106	0.2743	0.1308	0.304
Uniformity	μm	0.248	3.09	0.65	11.76
Homogeneity	μm	0.0184	0.2278	0.0295	0.4041

* GPD = Gold Pattern Diagnosis.

** μm (micrometer) = 1 x 10^-6^m.

### Processing and classification of data – Fuzzy Method

Before the proposed problem involving various fuzzy situations and considering the literature studied, it was found that the strategy of applying fuzzy logic could bring greater benefits (like expert knowledge acquisition, rules base generation, process automation and pre diagnosis greater precision) and satisfactory results, in addition to dealing with modelling, representation, the reasoning and the inaccurate information procedure as a troubleshooting strategy.

Thus, the implementation of the intervention and control actions in the intelligent method developed, uses fuzzy logic since it enables to capture the experts’ knowledge, as well as the appropriate treatment to fuzzy situations inherent in the problem classifying smears from breast mass obtained by FNA.

The algorithm developed to assist the creation of fuzzy system applied to the medical field is presented below.

**Algorithm:** establishment of fuzzy system applied to the medical area

Step 1: Definition

-> Identify the problem

Step 2: Medical knowledge acquisition

-> Obtain technical information from one or more medical specialists

-> Extract data and information from gold pattern databases (with diagnosis confirmed)

-> Obtain information in technical literature available

Step 3: Fuzzification stage

-> Define entry membership functions and their fuzzy rules

Step 4: Rules base

-> Define fuzzy rules covering all possibilities

Step 5: Inference Stage

-> Reporting observations to fuzzy sets

-> Evaluate each case for all fuzzy rules

-> Combine the information from the defined fuzzy rules

Step 6: Defuzzification stage

-> Define membership functions and output sets

-> Define the defuzzification function

Step 7: Results verification

-> Ask results are satisfactory?

If answer = “No”

-> Return to Step 2

If answer = “Yes”

Finalize

This way, the definition of Fuzzy Method to assist in the diagnosis of breast cancer and its stages (Fuzzification Stage, Rules Base, Inference Stage and Defuzzification Stage) are listed below and instantiated through the system implemented.

### PDM-FNA-Fuzzy Definition

Pre-Diagnosis Module FNA-Fuzzy performs the analysis of extracted descriptors of smears from breast mass obtained by FNA, considering the parameters that indicate malignant and benign diagnosis and the fuzzy rules base defined, responsible for inferences in the set of entries, generating pre-diagnosis, malignant or benign, to assist the diagnosis of breast cancer made by the doctor.

Experiments were carried out with all possible combinations of descriptors listed in Table 1, i.e. in addition to fuzzy methods for each descriptor, models have been developed for all groups of two, three, four, and so on up to the limit of nine descriptors, taking into account that the descriptors correspond to the input variables of fuzzy method. Within the set AREA, PERIMETER, UNIFORMITY and HOMOGENEITY produced the best results, the PDM-FNA-Fuzzy in question uses these four descriptors, with fuzzy method as described below.

### Fuzzification Stage

**Figure 5:**
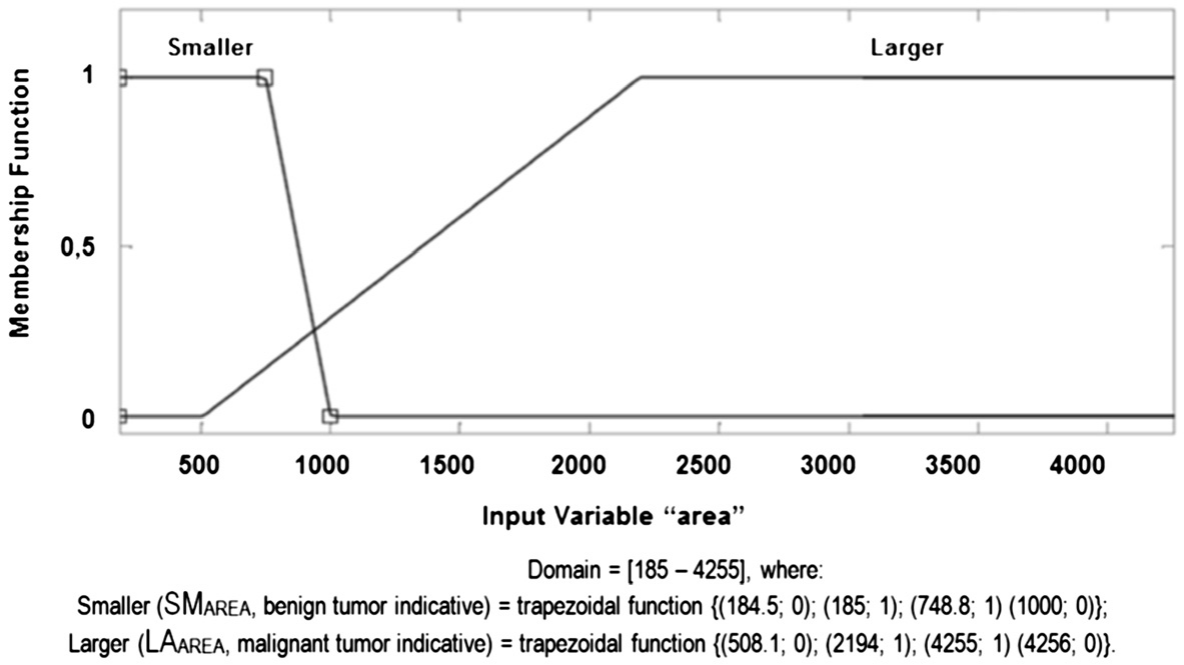
AREA Membership Function.

At this stage the input variables have been defined, identifying to which fuzzy sets they belong, assigning the respective degree to each relevance. The fuzzy sets, represented by the membership functions, were adjusted by heuristics, on the universe of discourse in order to improve the results achieved. Thus, before the creation of the fuzzy system, it was necessary to define the membership functions (fuzzy sets) used both at the fuzzification and defuzzification stages. The entries of PDM-FNA-Fuzzy in question are the descriptors AREA, PERIMETER, UNIFORMITY and HOMOGENENEITY that have been defined through the membership functions described below:

a. **Area membership function (AREA):** considering a domain of [185 – 4255], this membership function is composed of "Smaller AREA" and "Larger AREA", in linguistic terms SM_AREA_ and LA_AREA_, respectively, representing the tracks, according to the fuzzy set below and illustrated in Figure 5.

**Figure 6:**
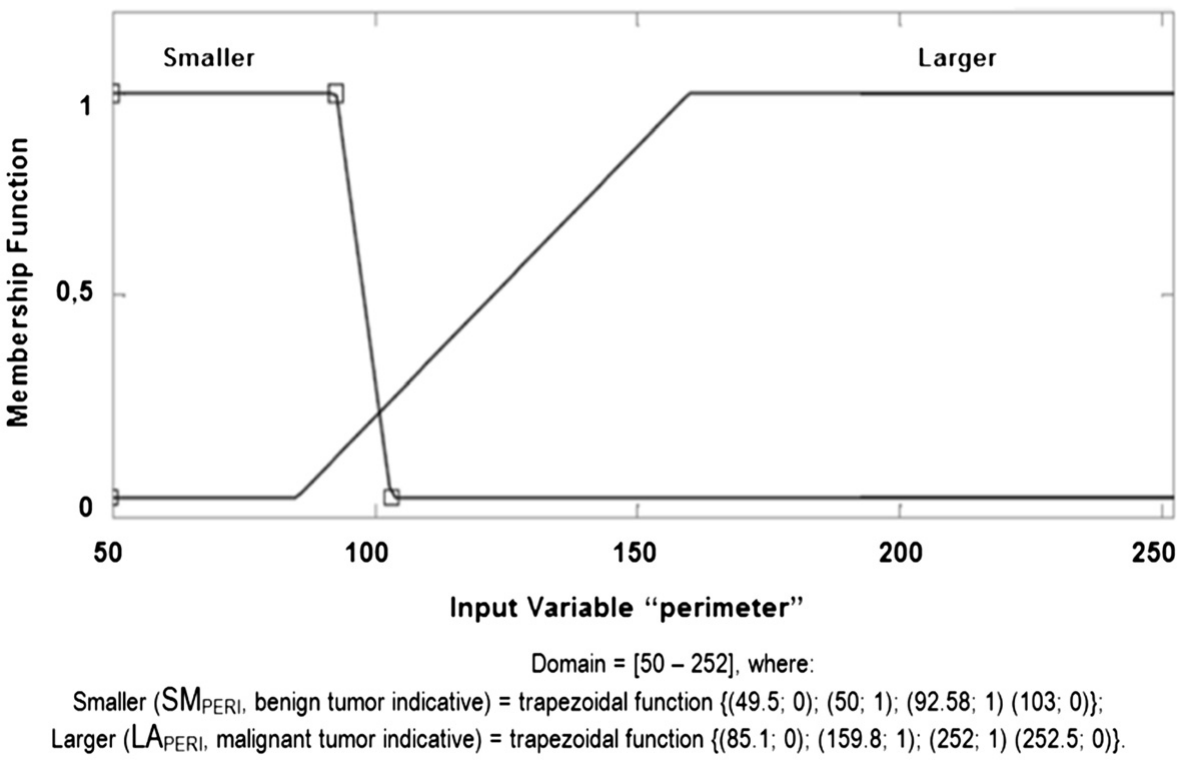
PERIMETER Membership Function.

AREA fuzzy set:

$$\begin{array}{l} \text{Smaller}\;\text{AREA}\;\left({\text{SM}}_{\text{AREA}}\right)\leq 100\to {\text{SM}}_{\text{AREA}}=\left\{\left(184.5;0\right),\left(185;1\right),\left(748.8;1\right),\left(100;0\right)\right\};\\ \text{Lager}\;\text{AREA}\;\left({\text{LA}}_{\text{AREA}}\right)\geq 508.1\to {\text{LA}}_{\text{AREA}}=\left\{\left(508.1;0\right),\left(219;1\right),\left(4255;1\right),\left(4256;0\right)\right\}\text{.} \end{array}$$

b. **Perimeter membership function (PERI):** considering a domain of [50 – 252], this membership function is composed of "Smaller PERI" and "Larger PERI", in linguistic terms SM_PERI_ and LA_PERI_, respectively, representing the tracks, according to the fuzzy set below and illustrated in Figure 6.

**Figure 7:**
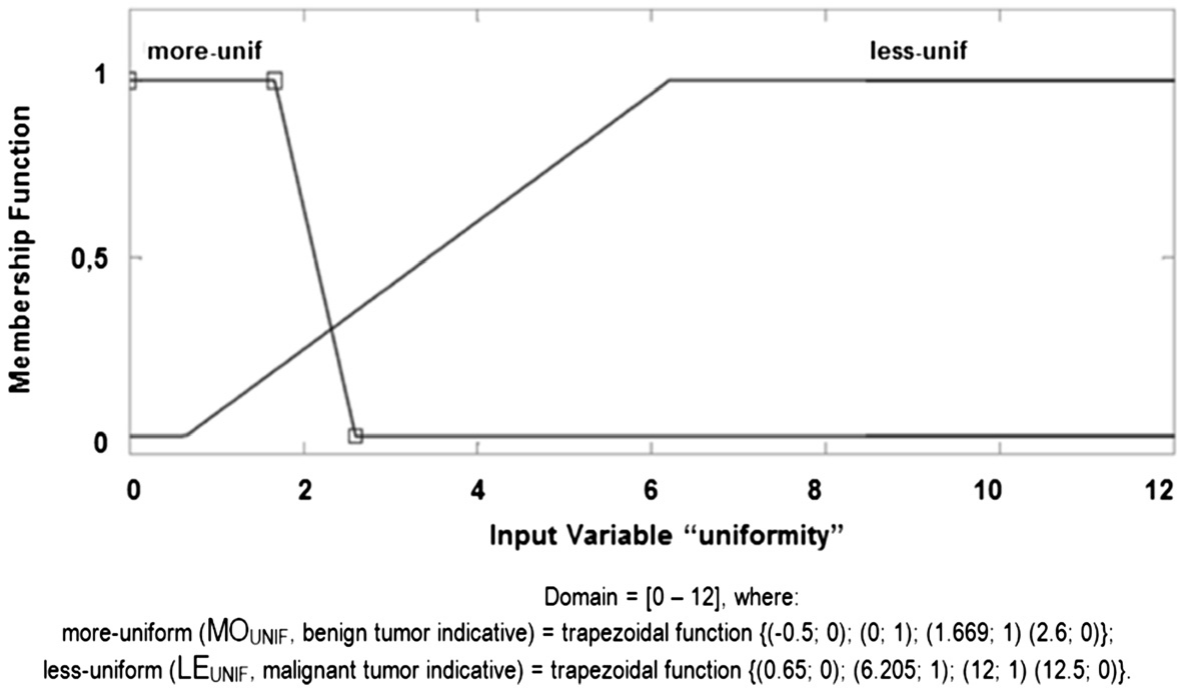
UNIFORMITY Membership Function.

PERIMETER fuzzy set:

$$\begin{array}{l} \text{Smaller}\;\text{PERI}\;\left({\text{SM}}_{\text{PERI}}\right)\leq \;103\to {\text{SM}}_{\text{PERI}}=\left\{\left(49.5;0\right),\left(50;1\right),\left(92.58;1\right),\left(103;0\right)\right\};\\ \text{Larger}\;\text{PERI}\;\left({\text{LA}}_{\text{PERI}}\right)\geq \;85.1\to {\text{LA}}_{\text{PERI}}=\left\{\left(85.1;0\right),\left(159.8;1\right),\left(252;1\right),\left(252.5;0\right)\right\}\text{.} \end{array}$$

c. **Uniformity membership function (UNIF):** considering a domain of [0 – 12], this membership function is composed of "More UNIF" and "Less UNIF", linguistically represented as MO_UNIF_ and LE_UNIF_, respectively, representing the tracks, according to the fuzzy set below and illustrated in Figure 7. It is important to note that, for this descriptor, more UNIF is associated with lower values (i.e. smaller values in this descriptor indicate there is more uniformity among the cellular nuclei and indicate a benign diagnosis) and less UNIF is associated to larger values (i.e. the larger values in this descriptor indicate there is less uniformity and indicate malignant diagnosis).

**Figure 8:**
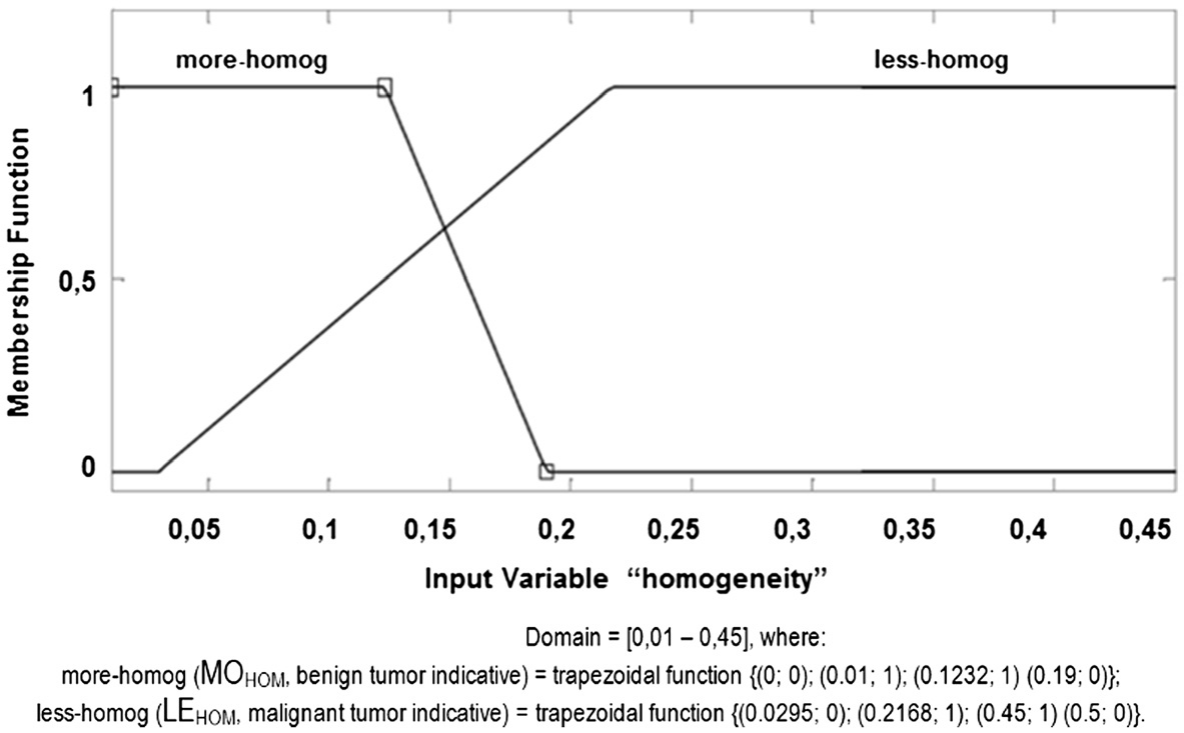
HOMOGENEITY Membership Function.

UNIFORMITY fuzzy set:

$$\begin{array}{l} \text{More}\;\text{UNIF}\;\left({\text{MO}}_{\text{UNIF}}\right)\leq \;2.6\to {\text{MO}}_{\text{UNIF}}=\left\{\left(-0.5;0\right),\left(0;1\right),\left(1.669;1\right),\left(2.6;0\right)\right\};\\ \text{Less}\;\text{UNIF}\;\left({\text{LE}}_{\text{UNIF}}\right)\geq 0.65\to {\text{LE}}_{\text{UNIF}}=\left\{\left(0.65;0\right),\left(6.205;1\right),\left(12;1\right),\left(12.5;0\right)\right\}\text{.} \end{array}$$

d. **Homogeneity membership function (HOM):** considering a domain of [0.01 – 0.45], this membership function is composed of "More HOM" and "Less HOM", in linguistic terms MO_HOM_ and LE_HOM_, respectively, representing the tracks, according to the fuzzy set below and illustrated in Figure 8. It is important to note that, for this descriptor, more HOM is associated with lower values (i.e. smaller values in this descriptor indicate there is more homogeneity among the cellular nuclei and indicate a benign diagnosis) and less HOM is linked to larger values (i.e. larger values in this descriptor indicate there is less homogeneity and indicate a diagnosis of malignancy).

HOMOGENEITY fuzzy set:

$$\begin{array}{l} \text{More}\;\text{HOM}\;\left({\text{MO}}_{\text{HOM}}\right)\leq 0.19\text{→}{\text{MO}}_{\text{HOM}}=\left\{\left(0;0\right),\left(0.01;1\right),\left(0.1232;1\right),\left(0.19;0\right)\right\};\\ \text{Less}\;\text{HOM}\;\left({\text{LE}}_{\text{HOM}}\right)\geq 0.0295\text{→}{\text{LE}}_{\text{HOM}}=\left\{\left(0.0295;0\right),\left(0.2168;1\right),\left(0.45;1\right),\left(0.5;0\right)\right\}\text{.} \end{array}$$

The membership functions were built using the direct method, having been confirmed by the medical experts (in pathology, mastology and general practice) the parameters extracted from WDBC, covering all data of the membership functions (values that represent each function and the degree of relevance, within the function, of each one of them) in order to set them explicitly. There are several membership functions that can be used at this fuzzification stage. All functions available in Matlab were applied (trials and tests) on the fuzzy system concerned, noting that the trapezoidal function was the one that presented the best results in PDM-FNA-Fuzzy, by best representing the functions according to the context.

### Rules Base – Fuzzy Rules definition

The rules base was assembled with the following structure: IF <premises>THEN <conclusion>. For the definition of rules of PDM-FNA-Fuzzy to assist in the diagnosis of breast cancer in question, it is possible to standardize the following structure:

$$\begin{array}{l} R:\left\{R_{1},R_{2},R_{3},\ldots ,R_{n}\right\}\text{→}\text{Set of rules};\\ \text{DESC}:\left\{{\text{DESC}}_{1},{\text{DESC}}_{2},{\text{DESC}}_{3},\ldots ,{\text{DESC}}_{n},\right\}\text{→}\text{Set of descriptors}\;\\ \;\left(\text{representing the set of entries}\right);\\ P:\{B(\text{↓}),\;\text{Undef}(\text{↓}\text{↑}),\;M(\text{↑})\}\text{→}\text{Parameterization of the descriptor’s situation}\\ \;\left(\text{Benign},\text{Undefined and Malignant}\right);\\ D:\left\{D_{1},D_{2},D_{3},\ldots ,D_{n}\right\}\text{→}\text{Set of diagnosis possibilities}\text{.} \end{array}$$

Rules definition:

$$\begin{array}{l} <\left(R_{1},R_{2},R_{3},\ldots ,R_{n},\right)>:IF<\left(\mathrm{DESC}1,\mathrm{DESC}2,\mathrm{DESC}3,\ldots ,\mathrm{DESCn},\right)>\\ <\{\;B\;(\text{↓}),\text{Undef}(\text{↓}\text{↑}),\;M\;(\text{↑})\}>\text{and}/\text{or}<\left(\mathrm{DESC}1,\mathrm{DESC}2,\mathrm{DESC}3,\ldots ,\mathrm{DESCn},\right)>\\ <\{\;B\;(\text{↓}),\text{Undef}(\text{↓}\text{↑}),\;M\;(\text{↑})\}>\text{and}/\text{or}\ldots \text{THEN}<D_{1},{\;D}_{2},{\;D}_{3},\ldots ,{\;D}_{\text{n}}> \end{array}$$

Consequently, 16 (sixteen) rules were defined for PDM-FNA-Fuzzy object of this study, using 4 (four) descriptors and with 3 (three) possibilities of pre-diagnosis <results>. To exemplify, below some of the rules:

**# Rule 1:** If smaller AREA and smaller PERI and more UNIF (descriptor with lower value) and more HOM (descriptor with smaller value) then benign diagnosis.

ifAREA↓andPERI↓andUNIF↓andHOM↓thenB

**# Rule13:** If larger AREA and larger PERI and more UNIF (descriptor with lower value) and more HOM (descriptor with smaller value) then diagnosis undefined.

ifAREA↑andPERI↑andUNIF↓andHOM↓thenUndef

**# Rule 16:** If larger AREA and larger PERI and less UNIF (understand descriptor with larger value) and less HOM (descriptor with larger value) then malignant diagnosis.

ifAREA↑andPERI↑andUNIF↑andHOM↑thenM

We must point out that the rules defined (16 rules) cover all possible combinations of inputs and outputs of the proposed issue and that the consistency of the rules was examined in order to avoid contradictions. The rules base, presented in the Table 2, was developed from the analysis of numerical data and multiple meetings, discussions and interviews with medical experts from the fields of pathology, mastology, and general practitioners.

**Table 2:** Rules base

**Rule number**	**Rule specification**
1	**if** AREA ↓ **and** PERI ↓ **and** UNIF ↓ **and** HOM ↓ **then** B
2	**if** AREA ↓ **and** PERI ↓ **and** UNIF ↓ **and** HOM ↑ **then** Undef
3	**if** AREA ↓ **and** PERI ↓ **and** UNIF ↑ **and** HOM ↓ **then** Undef
4	**if** AREA ↓ **and** PERI ↓ **and** UNIF ↑ **and** HOM ↑ **then** Undef
5	**if** AREA ↓ **and** PERI ↑ **and** UNIF ↓ **and** HOM ↓ **then** Undef
6	**if** AREA ↓ **and** PERI ↑ **and** UNIF ↓ **and** HOM ↑ **then** Undef
7	**if** AREA ↓ **and** PERI ↑ **and** UNIF ↑ **and** HOM ↓ **then** Undef
8	**if** AREA ↓ **and** PERI ↑ **and** UNIF ↑ **and** HOM ↑ **then** Undef
9	**if** AREA ↑ **and** PERI ↓ **and** UNIF ↓ **and** HOM ↓ **then** Undef
10	**if** AREA ↑ **and** PERI ↓ **and** UNIF ↓ **and** HOM ↑ **then** Undef
11	**if** AREA ↑ **and** PERI ↓ **and** UNIF ↑ **and** HOM ↓ **then** Undef
12	**if** AREA ↑ **and** PERI ↓ **and** UNIF ↑ **and** HOM ↑ **then** Undef
13	**if** AREA ↑ **and** PERI ↑ **and** UNIF ↓ **and** HOM ↓ **then** Undef
14	**if** AREA ↑ **and** PERI ↑ **and** UNIF ↓ **and** HOM ↑ **then** Undef
15	**if** AREA ↑ **and** PERI ↑ **and** UNIF ↑ **and** HOM ↓ **then** Undef
16	**if** AREA ↑ **and** PERI ↑ **and** UNIF ↑ **and** HOM ↑ **then** M

It should be noted that, following the medical practice, the procedure taken for undefined (Undef) pre-diagnosis (results) are referred to as the situation of suspected malignant tumour, which indicates a biopsy procedure(similar to the malignant pre-diagnosis), i.e., if in doubt the patient is referred for a biopsy. Thus, from the classification point of view for having a biopsy or not, the PDM-FNA-Fuzzy can be seen as a binary classifier with the record of 2.1% of cases classified as undefined and thus regarded as malignant.

### Inference stage

In this stage, the entries were analysed to generate the fuzzy output set with its respective compatibility degree. The PDM-FNA-Fuzzy developed used the fuzzy model proposed by Mamdani [29], in which the activation function of each rule is enabled and the system of inference determines the degree of compatibility of the rules premise contained in the rules base. After this, it determines which rules are enabled and applies them to the output membership function, remaining just linking all output nebulous sets activated (and their respective degrees of compatibility) into a single Output Set (OS). This OS represents all results (diagnosis) that are acceptable for the input set, each with its compatibility level. Each case was also assessed, at this stage, for all fuzzy rules and the combination of information was carried out from the rules already defined in the Rules Base.

### Defuzzification stage

This stage was used to generate a single numeric value, from all possible values contained in the fuzzy set obtained in the inference stage, to generate the diagnosis. As a diagnosis resulting from the relations and variability of the descriptors AREA, PERIMETER, UNIFORMITY and HOMOGENEITY, the function centroid (which presented the best results) and the domain [0 – 1] was adopted for defuzzification.

**Figure 9:**
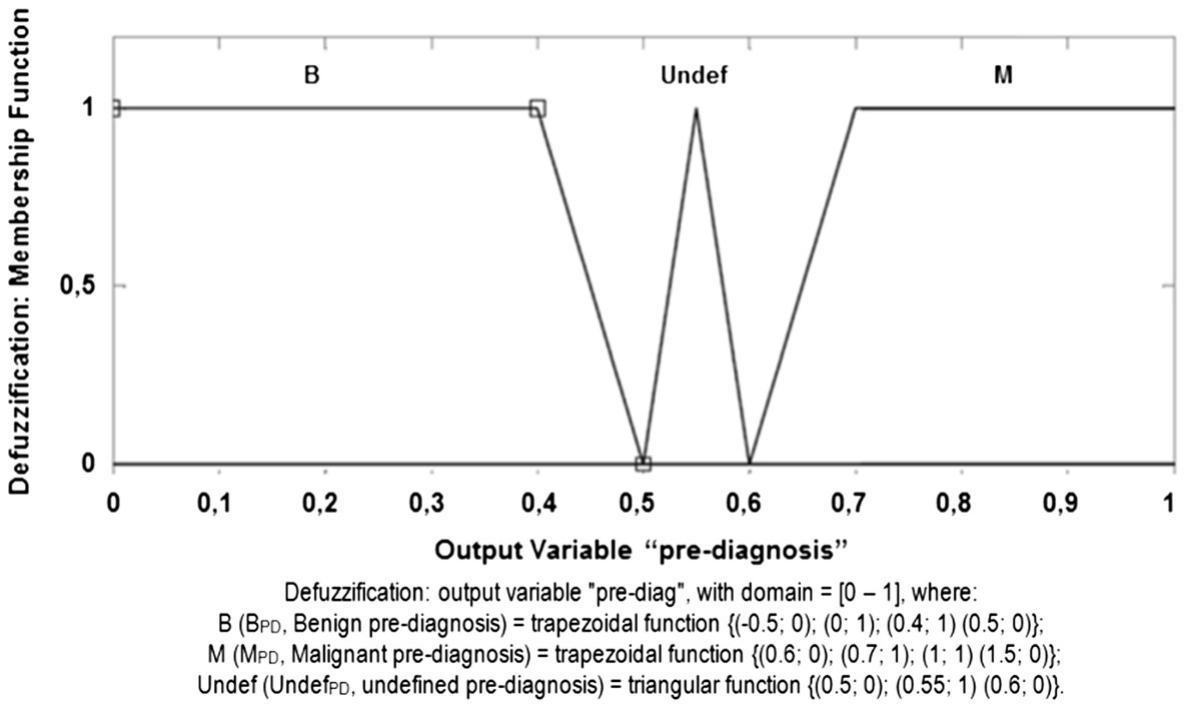
Defuzzification Membership Function.

The Pre-Diagnosis (PD) membership function, Defuzzification, is composed of "Benign", "Undefined" and "Malignant", represented linguistically as B_PD_, Undef_PD_ and M_PD_, respectively, representing the tracks [≤ 0.5; 0.5 – 0.6; and ≥ 0.6], as output set below and illustrated in Figure 9.

Output Set (OS):

$$\begin{array}{l} \text{PD benign}\;\left(B_{\text{PD}}\right)\leq 0.5\text{→}B_{\text{PD}}=\left\{\left(-0.5;0\right),\left(0;1\right),\left(0.4;1\right),\left(0.5;0\right)\right\};\\ \text{PD undefined}\;\left({\text{Undef}}_{\text{PD}}\right)\geq 0.5\;e\;\leq 0.6\text{→}{\text{Undef}}_{\text{PD}}=\left\{\left(0.5;0\right),\left(0.55;1\right),\left(0.6;0\right)\right\};\\ \text{PD malignant}\;\left(M_{\text{PD}}\right)\geq 0.6\text{→}M_{\text{PD}}=\left\{\left(0.6;0\right),\left(0.7;1\right),\left(1;1\right),\left(1.5;0\right)\right\}\text{.} \end{array}$$

### Post-processing

In post-processing, the result, in the form of malignant or benign pre-diagnosis, is stored on the server and made available on the screen by means of IVEMI (on the desktop discussion of clinical case), both for the doctor who requested the pre-diagnosis or second opinion, as to the other users with access permission to the respective clinical case.

### Validation

Testing of the PDM-FNA-Fuzzy, the object of this study, was carried out using the MATLAB R2010a (student version), due to the tools available in this application to the development of models and the rapid visualization of the results obtained in the fuzzy system.

The PDM-FNA-Fuzzy developed to assist in the diagnosis of breast cancer performs the interaction between the descriptors AREA, PERIMETER, UNIFORMITY and HOMOGENEITY (extracted from smears obtained by FNA), operated by inference rules of the system expert in fuzzy logic, triggering classification and assistance in medical diagnosis actions.

All the tests were initially carried out using the WDBC, even the identification of the main characteristics of the fuzzy system, such as:

the identification of the set of descriptors that provide the best results, called "best input set" (BIS);

identification of the best set of rules (BSR); and

the definition of what membership functions, which parameters and what defuzzification functions are most suitable for use with the BIS and the BSR.

The membership functions and their respective fuzzy final sets of each descriptor used, AREA, PERIMETER, UNIFORMITY and HOMOGENEITY, are presented, respectively, in Figures 5, 6, 7 and 8.

The validation of the rules base was held in conjunction with medical professionals (in pathology, general practice and mastology), considering the fuzzy set indicators of both malignant and benign diagnosis. As a consequent action of the descriptors’ relations and variability the domain [0 –1], representing the tracks [< 0.5; 0.5 – 0.6; > 0.6], was adopted to defuzzification, which is represented in linguistic terms as “Benign”, “Undefined” and “Malignant”, respectively, as presented in Figure 9.

After this phase, cross-validation was used for testing, in order to fine-tune the parameters of the membership functions of PDM-FNA-Fuzzy. Therefore, three databases were generated, each of them with 150 (one hundred and fifty) gold pattern clinical cases randomly extracted from WDBC.

The validation of PDM-FNA-Fuzzy was performed using a database with 100 (one hundred) gold pattern clinical cases (i.e. diagnosis known and confirmed), randomly extracted from WDBC.

We must point out that the validations of both the knowledge gained and the results achieved were performed during the development of PDM-FNA-Fuzzy and also, in the final instance, by medical specialists in the areas of pathology, mastology and general practice.

## Results

By means of the experiments performed, it was found that the input set that featured the best results has the following characteristics:

a) **fuzzy system:** Mamdani;

b) **membership functions of the entry set:** trapezoidal;

c) **input set** composed of 4 variables (descriptors), with the following fuzzy sets:

c.1)

$$\begin{array}{l} \text{AREA}\;\text{com}\;{\text{SM}}_{\text{AREA}}=\left\{\left(184.5;0\right),\left(185;1\right),\left(748.8;1\right),\left(1000;0\right)\right\}\\ \;\text{and}\;{\text{LA}}_{\text{AREA}}=\left\{\left(508.1;0\right),\left(2194;1\right),\left(4255;1\right),\left(4256;0\right)\right\}\text{;} \end{array}$$

c.2.)

$$\begin{array}{l} \text{PERIMETER}\;{\text{withL}}_{\text{PERI}}=\left\{\left(49.5;0\right),\left(50;1\right),\left(92.58;1\right),\left(103;0\right)\right\}\;\\ \text{and}\;{\text{LA}}_{\text{PERI}}=\left\{\left(85.1;0\right),\left(159.8;1\right),\left(252;1\right),\left(252.5;0\right)\right\}\text{;} \end{array}$$

c.3.)

$$\begin{array}{l} \text{UNIFORMITY}\;\text{with}\;{\text{MO}}_{\text{UNIF}}=\left\{\left(-0.5;0\right),\left(0;1\right),\left(1.669;1\right),\left(2.6;0\right)\right\}\;\\ \text{and}\;{\text{LE}}_{\text{UNIF}}=\left\{\left(0.65;0\right),\left(6.205;1\right),\left(12;1\right),\left(12.5;0\right)\right\};\;\text{and} \end{array}$$

c.4.)

$$\begin{array}{l} \text{HOMOGENEITY}\;\text{with}\;{\text{MO}}_{\text{HOM}}=\left\{\left(0;0\right),\left(0.01;1\right),\left(0.1232;1\right),\left(0.19;0\right)\right\}\;\\ \text{and}\;{\text{LE}}_{\text{HOM}}=\left\{\left(0.0295;0\right),\left(0.2168;1\right),\left(0.45;1\right),\left(0.5;0\right)\right\}\text{;} \end{array}$$

d) **rules base:** 16 rules;

e) membership functions of the output set:

e.1)

$$\begin{array}{l} \text{trapezoidal for classification}\text{Benign},\text{being}\;B_{\text{PD}}\\ \;=\left\{\left(-0.5;0\right),\left(0;1\right),\left(0.4;1\right),\left(0.5;0\right)\right\}\text{;} \end{array}$$

e.2)

$$\begin{array}{l} \text{trapezoidal for classification}\text{Malignant},\text{being}\;M_{\text{PD}}\\ \;=\left\{\left(0.6;0\right),\left(0.7;1\right),\left(1;1\right),\left(1.5;0\right)\right\};\text{and} \end{array}$$

e.3)

$$\begin{array}{l} \text{triangular for classification}\text{Undefined},\text{being}\;{\text{Undef}}_{\text{PD}}\\ \;=\left\{\left(0.5;0\right),\left(0.55;1\right),\left(0.6;0\right)\right\}\text{;} \end{array}$$

f) **defuzzification:** Centroid function;

g) **output variable:** 1 (result = pre-diagnosis).

The best result achieved is shown in the Diagnostic Test Assessment Matrix presented in Table 3, as well as in the Matrix of Confusion presented in Table 4.

**Table 3:** Diagnostic test of assessment matrix of PDM-FNA-Fuzzy developed to assist in the diagnosis of breast cancer

**Diagnostic test Assessment**			
	**GOLD PATTERN DIAGNOSIS**		
**FUZZY-FNA**	Malignant (%)	Benign (%)	**TOTAL**
Malignant (%)	36.73	9.14	45.87
Benign (%)	0.53	53.60	54.13
**TOTAL**	37.26	62.74	100.00
**Sensitivity = 98.59%**	**Specificity = 85.43%**		

**Table 4:** Confusion matrix of the diagnostic test of PDM-FNA-Fuzzy developed to assist the diagnosis of breast cancer

**Confusion matrix**		
	**GOLD PATTERN**	
**FUZZY-FNA**	Malignant	Benign
Malignant	**0.99**	0.15
Benign	0.01	**0.85**

It is noted in the diagnostic test assessment matrix (Table 3), that the PDM-FNA-Fuzzy developed presents: 98.59% sensitivity, which is the ability of a diagnostic test to identify the real positive in individuals truly ill, meaning a satisfactory percentage of hits in the pre-diagnosis of malignancies; and 85.43% specificity, which is the ability of a diagnostic test to identify the real negative in individuals truly healthy, corresponding to the correct pre-diagnosis of benign cases.

We must point out that, in the laboratory examination (biopsy) of smears obtained by FNA for identification of breast cancer, it is more important to get good results in sensitivity than in specificity ([30-32]). Subsequently, among the tests performed during the development of PDM-FNA-Fuzzy to assist in the diagnosis of breast cancer, there were several with satisfactory results as well, but they were not selected as the best solution, having been discarded, as, for example, the test sets A, B and C, presented below.

The tests of set A were conducted from the best input set, with changes in nebulous sets (parameters) of the membership functions. In Table 5, the results of sensitivity and specificity of the same are presented. Notably test A.1 presents 99.06% sensitivity, however the medical experts found the specificity of 64.15% unsatisfactory. The tests A.8 and A.10 presented the same sensitivity of PDM-FNA-Fuzzy developed (98.59%), but lower specificity (84.31% and 84.87%, respectively). The other tests presented sensitivity less than 98.59% and thus were discarded.

**Table 5:** Comparison of the tests presented in “TEST SET A" (changes were realized in the fuzzy sets of membership functions)

**Tests**	**Sensitivity (%)**	**Specificity (%)**
**PDM-FNA-Fuzzy developed**	**98.59**	**85.43**
Test A.1 ^(1)^	99.06	64.15
Test A.2 ^(2)^	92.92	90.48
Test A.3 ^(3)^	98.11	70.87
Test A.4 ^(4)^	93.87	89.92
Test A.5 ^(5)^	96.23	88.80
Test A.6 ^(6)^	97.17	87.39
Test A.7 ^(7)^	97.64	86.83
Test A.8 ^(8)^	98.59	84.31
Test A.9 ^(9)^	98.11	86.55
Test A.10 ^(10)^	98.59	84.87

(1) changes in Test A.1: SM_AREA_ = {(184.5; 0), (185; 1), (749; 1), (800; 0)} e SM_PERI_ = {(49.5; 0), (50; 1), (92.6; 1), (95; 0)} e MO_UNIF_ = {(-0.5; 0), (0; 1), (1.67; 1), (1.87; 0)} e MO_HOM_ = {(0; 0), (0.01; 1), (0.123; 1), (0.143; 0)}.

(2) changes in Test A.2: SM_AREA_ = {(184.5; 0), (185; 1), (748.8; 1), (800; 0)} e SM_PERI_ = {(49.5; 0), (50; 1), (92.58; 1), (127.1; 0)} e MO_UNIF_ = {(-0.5; 0), (0; 1), (1.669; 1), (3.09; 0)} e MO_HOM_ = {(0; 0), (0.01; 1), (0.1232; 1), (0.2278; 0)}.

(3) changes in Test A.3: SM_AREA_ = {(184.5; 0), (185; 1), (748.8; 1), (800; 0)} e SM_PERI_ = {(49.5; 0), (50; 1), (92.58; 1), (95; 0)} e MO_UNIF_ = {(-0.5; 0), (0; 1), (1.669; 1), (3.09; 0)} e MO_HOM_ = {(0; 0), (0.01; 1), (0.1232; 1), (0.2278; 0)}.

(4) changes in Test A.4: SM_AREA_ = {(184.5; 0), (185; 1), (748.8; 1), (800; 0)} e SM_PERI_ = {(49.5; 0), (50; 1), (92.58; 1), (110; 0)} e MO_UNIF_ = {(-0.5; 0), (0; 1), (1.669; 1), (3.09; 0)} e MO_HOM_ = {(0; 0), (0.01; 1), (0.1232; 1), (0.2278; 0)}.

(5) changes in Test A.5: SM_AREA_ = {(184.5; 0), (185; 1), (748.8; 1), (800; 0)} e SM_PERI_ = {(49.5; 0), (50; 1), (92.58; 1), (106; 0)} e MO_UNIF_ = {(-0.5; 0), (0; 1), (1.669; 1), (3.09; 0)} e MO_HOM_ = {(0; 0), (0.01; 1), (0.1232; 1), (0.2278; 0)}.

(6) changes in Test A.6: SM_AREA_ = {(184.5; 0), (185; 1), (748.8; 1), (800; 0)} e SM_PERI_ = {(49.5; 0), (50; 1), (92.58; 1), (106; 0)} e MO_UNIF_ = {(-0.5; 0), (0; 1), (1.669; 1), (3.09; 0)} e MO_HOM_ = {(0; 0), (0.01; 1), (0.1232; 1), (0.18; 0)}.

(7) changes in Test A.7: SM_AREA_ = {(184.5; 0), (185; 1), (748.8; 1), (800; 0)} e SM_PERI_ = {(49.5; 0), (50; 1), (92.58; 1), (106; 0)} e MO_UNIF_ = {(-0.5; 0), (0; 1), (1.669; 1), (2.5; 0)} e MO_HOM_ = {(0; 0), (0.01; 1), (0.1232; 1), (0.18; 0)}.

(8) changes in Test A.8: SM_AREA_ = {(184.5; 0), (185; 1), (748.8; 1), (800; 0)} e MO_UNIF_ = {(-0.5; 0), (0; 1), (1.669; 1), (2.5; 0)} e MO_HOM_ = {(0; 0), (0.01; 1), (0.1232; 1), (0.18; 0)}.

(9) changes in Test A.9: SM_PERI_ = {(49.5; 0), (50; 1), (92.58; 1), (103.5; 0)}.

(10) changes in Test A.10: SM_PERI_ = {(49.5; 0), (50; 1), (92.58; 1), (102.3; 0)}.

The tests of set B were conducted from the best input set, with changes in the types of membership function of the input set and, consequently, in their nebulous set (parameters). In Table 6, the results of sensitivity and specificity of the same are presented. Notably the tests B.1 and B.4 showed the same sensitivity that the PDM-FNA-Fuzzy developed (98.59%), but lower specificity (84.47% and 82.91%, respectively). The other tests showed sensitivity less than 98.59%, having been discarded.

**Table 6:** Comparison of the tests presented in "TEST SET B" (changes were realized in the membership functions of the entry set and its fuzzy sets)

**Tests**	**Type of membership function (after adjustments in fuzzy sets)**	**Sensitivity (%)**	**Specificity (%)**
**PDM-FNA-Fuzzy developed**	**trapezoidal**^**(1)**^	**98.59**	**85.43**
Test B.1	triangular^(2)^	98.59	83.47
Test B.2	gaussian2^(3)^	98.11	84.31
Test B.3	dsigmoidal^(4)^	98.11	84.59
Test B.4	polinomial zero^(5)^	98.59	82.91

(1) trapezoidal - function with straight lines with a flat top, resembling a truncated triangle.

(2) triangular - function with straight lines, in the form of a triangle.

(3) gaussiana2 - composed of two different gaussian curves.

(4) dsigmoidal - created from the difference between two sigmoidais functions.

(5) polinomial zero – asymmetric polynomial function, being zero at both ends, with an increase in the middle.

The C set were performed from the best input set, with changes only in the defuzzification function. Presented in Table 7, are the results of sensitivity and specificity of the same. It is worthy to note that all of the tests presented the same sensitivity that the PDM-FNA-Fuzzy developed (98.59%), but lower specificity.

**Table 7:** Comparison of tests presented in "TEST SET C" (changes were realized in the defuzzification functions)

**Tests**	**Defuzzification function**	**Sensitivity (%)**	**Specificity (%)**
**PDM-FNA-Fuzzy developed**	**centroid**^**(1)**^	**98.59**	**85.43**
Test C.1	bisector^(2)^	98.59	83.47
Test C.2	mom^(3)^	98.59	77.59
Test C.3	lom^(4)^	98.59	73.67
Test C.4	som^(5)^	98.59	77.59

(1) centroid - calculates the output set (OS) area center generated in the inference stage and determines its projection on the x-axis, that is the control output value.

(2) bisector - exact position that splits the output set into two equal areas.

(3) mom (Middle of Maximum) - it performs the arithmetic mean of all maximum values of the OS.

(4) lom (Largest of Maximum) - considers the greatest among all the maximum values of the OS.

(5) som (Smallest of Maximum) - considers the lowest among all the maximum values of the OS.

Thus, the results achieved by the PDM-FNA-Fuzzy, the object of this study, were considered satisfactory by medical specialists (in pathology, general practice and mastology), mainly for their high sensitivity (malignant cases hit) presented, as can be seen in Table 3.

The sensitivity of 98.59% presented by MPD-FNA-Fuzzy is at the same level of prominence of other works using the same dataset with other techniques such as, for example, [11] and [14], using Probabilistic Neural Network-PNN with 31-568-2 topology. Although other works, for example, [11], [12][14], [15], are more accurate than MPD-FNA-Fuzzy, they use ten descriptors, while the MPD-FNA-Fuzzy uses only four descriptors, two of which are extracted indirectly from WDBC, which simplifies the model and streamlines processing.

## Conclusions

This work presented an intelligent method to assist the diagnosis and second opinion of breast cancer, using a fuzzy method capable of processing and sorting data (descriptors) extracted from smears of breast mass obtained by FNA.

Processing, testing and validation using fuzzy method were carried out by medical specialists using the gold pattern database, i.e. with real data and real and verified diagnosis.

The main contributions of this paper are:

• specification and implementation of fuzzy method (MPD-FNA-Fuzzy) that meets the requirements to assist breast cancer diagnosis, carried out using the analysis of real data and contact with experts;

• reduction of malignant cases variation hit when compared to visual interpretation currently applied in the diagnosis by FNA. While the MPD-FNA-Fuzzy features stable sensitivity in 98.59%, visual interpretation diagnosis provides a sensitivity variation from 65% to 98%, this track showing sensitivity levels below those considered satisfactory by medical specialists;

• the use of intelligent systems techniques, more specifically, fuzzy logic, to assist the diagnosis and second opinion of breast cancer from smears of FNA;

• development of a Pre-Diagnosis Method that can be embedded into a virtual environment of medical interaction;

• detection of the main descriptors of WDBC to assist the diagnosis of breast cancer;

• creation, from the WDBC, of new important descriptors to assist the breast cancer diagnosis: UNIFORMITY and HOMOGENEITY;

• definition of algorithm for fuzzy system development applied to the medical field.

### Endnotes

^1^Gold pattern means that the true diagnosis is known and confirmed for each clinical case. In the case of WDBC, malignant diagnoses were confirmed by surgical biopsy and benign diagnosis by subsequent periodic medical examinations.

^2^Onofre Lopes Hospital (UFRN); Graduate Program in Health Sciences (UFRN); Promater Hospital; e Oncology and Mastology Clinic of Natal/RN.

^3^PCA (Principal Component Analysis) is a linear projection technique that performs statistical analysis of correlation between parameters, reducing the dimensionality of the problem [33].

^4^SOM (Self Organizing Map), also known as Kohonen self-organizing maps, that have the ability to form mappings that preserve the topology between input and output spaces [33].

## Competing Interests

The authors declare that they have no competing interests.

## Authors' contributions

The main contributions of the authors are as follows: GRMAS was responsible for the mapping and analysis of the descriptors WDBC, and also proposed the creation and utilization of the new descriptors (uniformity and homogeneity), as well as led the contact with the medical experts and competed adjustments in membership functions parameters; CRML devised the algorithm for fuzzy system development applied to the medical area and assisted in the definition of the rules base; AMGG participated as co-advisor of the research group; GRMAS, ADDN and AMGG designed the study and ADDN participated as Advisor and coordinator of the research group. All the authors have read and approved the final manuscript.
